# Endothelial cell-specific molecule-1 as an invasiveness marker for pituitary null cell adenoma

**DOI:** 10.1186/s12902-019-0418-8

**Published:** 2019-08-27

**Authors:** Shousen Wang, Zhifeng Wu, Liangfeng Wei, Jianhe Zhang

**Affiliations:** 1 Department of Neurosurgery, Fuzhou 900 Hospital, Fujian Medical University, No.156 Xihuanbei Road, Fuzhou, 350025 People’s Republic of China; 2 Department of Neurosurgery, Fujian provincial Hospital, Chinese People’s Armed Police Forces, Fuzhou, 350025 People’s Republic of China

**Keywords:** Angiogenesis, Endothelial cell-specific molecule-1, Invasiveness, Pituitary adenoma, Null cell adenoma

## Abstract

**Background:**

Endothelial cell-specific molecule-1 (ESM-1) is a biomarker associated with tumor progression in pituitary adenoma. We specifically focused on one type of pituitary adenoma, namely null cell adenoma (NCA) and evaluated the relationship between invasion and ESM-1 expression in both vascular endothelial and adenoma tissues.

**Methods:**

Tissue samples from 94 patients with pituitary NCA were obtained through microscopic transsphenoidal resection. Tumor size and invasion were determined through preoperative magnetic resonance imaging. Immunohistochemical staining was performed to detect ESM-1 expression. ESM-1 index of ≥3 was defined as high expression.

**Results:**

Signs of invasion were observed in 46 (47.9%) of the 94 patients. Significant differences were observed in the invasion state and maximum tumor diameter between high and low expression of ESM-1 in vascular endothelial tissues (both *P* < 0.05). Significant positive associations were noted between ESM-1 expression in vascular endothelial tissues and tumor invasion (*P* = 0.002) and tumor size (*P* = 0.020). However, only tumor size was associated with ESM-1 expression in adenoma tissues (*P* = 0.016).

**Conclusion:**

In NCA, a significant positive association between tumor invasion and ESM-1 expression was observed only in vascular endothelial tissues, suggesting that tumor progression occurs mainly through ESM-1-associated mechanism.

## Background

Endothelial cell-specific molecule-1 (ESM-1), also called endocan, is a dermatan sulfate proteoglycan secreted by endothelial cells [1, 2]. The production and secretion of ESM-1 are regulated by proangiogenic molecules, such as vascular endothelial growth factor (VEGF) and fibroblast growth factor (FGF) [3–5], both of which are possible markers of angiogenesis. Angiogenesis is critical for tumor initiation and progression in various tumors [6]. ESM-1 is a biomarker associated with tumor progression in various types of tumors, including lung, liver, brain, kidney, and stomach tumors, and ESM-1 overexpression has been associated with poor prognosis [7–11].

Whether angiogenesis plays a key role in tumor initiation and progression in pituitary adenoma remains controversial, and until now, mechanisms underlying angiogenesis in pituitary adenoma are also debatable. Some studies have found that several crucial biomarkers of angiogenesis, including VEGF and CD34, were widely expressed in pituitary adenoma [12, 13]; however, other studies have indicated that compared with the normal pituitary gland tissue, pituitary adenoma had lower angiogenesis and CD34/CD105 microvessel density (MVD) [14–17].

The relationship among angiogenesis, tumor initiation, and ESM-1 expression in pituitary adenoma should be investigated. In the normal pituitary gland tissue, ESM-1 expression was detected in isolated endocrine cells but not in vascular endothelial cells. By contrast, a strong association was found between ESM-1 immunoreactivity in vascular endothelial cells and progression in pituitary adenoma [18]. Through fluorescence immunohistochemistry, Matano et al. found that ESM-1 expression in pituitary adenoma was colocalized in more than 90% of CD34^+^ endothelial cells (i.e., vascular endothelial cells) and concluded that ESM-1 is related to tumor angiogenesis in pituitary adenoma [19]. By contrast, another study that examined tissues from 66 patients with pituitary adenoma reported that although both endothelial and tumor cells expressed ESM-1, ESM-1 expression was significantly higher in tumor cells than in endothelial cells, indicating that the biosynthesis and secretion of ESM-1 may mainly be from tumor cells in pituitary adenoma [14].

In addition to mechanisms underlying the involvement of ESM-1 in angiogenesis, the association between ESM-1 and invasion in pituitary adenoma remains unclear. No significant association was found in a study that included 107 patients [18], whereas ESM-1 expression has been associated with invasion represented by the Knosp grade in other studies [14, 19]. All the aforementioned findings indicate the uniqueness of pituitary adenoma, and additional investigations are urgently required to understand the role of ESM-1 in pituitary adenoma. In the present study, we specifically focused on one type of adenoma, namely null cell adenoma (NCA), and evaluated the relationship between invasion and ESM-1 expression both in vascular endothelial and adenoma tissues.

## Methods

### Patients

Tissue samples from 94 patients with pituitary NCA were obtained through resection performed using the microscopic transsphenoidal approach in our hospital between January 2010 and May 2014. The diagnosis of NCA was established on the basis of pathological, cytological, and immunochemical findings, according to the World Health Organization (WHO) classification of tumors (3rd edition, published in 2004) [20]. This study was approved the Institutional Review Board of Fuzhou 900 Hospital (No. 20140703).

Tumor size was determined through preoperative magnetic resonance imaging (MRI) by using the INFINITT PACS (INFINITT Healthcare, Seoul, Korea). Tumor invasion was evaluated through MRI and observation during surgery and was defined as meeting any one of the following criteria: Knosp grade 3 or 4 [21], Wilson–Hardy classification ≥IV or suprasellar extension grade C [22], or extrasellar extension.

### Immunohistochemical staining

Tissues were immediately fixed after surgery in 100 g/L formaldehyde, embedded in paraffin, and cut into 4-μm-thick sections. After deparaffinization and rehydration, antigen retrieval was performed in the sections by using 0.01 mol/L citric acid; the sections were then treated with 3% hydrogen peroxide for 10 min. The sections were blocked using 5–10% BSA and subsequently incubated with a primary monoclonal antibody, namely Ki-67 (1:100, MXB Biotechnology, Fujian, China) or ESM-1 (1:100, Bioss antibodies, Beijing, China), for 60 min at room temperature. The sections were then washed with PBS for 3–5 min three times and incubated for 15 min at room temperature with a horseradish peroxidase (HRP)-labeled secondary antibody that was included in the MaxVision HRP-conjugated Polymer Immunohistochemistry kit (MaxVision, Fujian, China). Color was developed through incubation with diaminobenzidine (Dako Corp, Carpinteria, CA, USA) at room temperature for 5–10 min, and the stained sections were observed under a light microscope by two experienced pathologists independently. The negative control was incubated with PBS, and human umbilical vein endothelial cells served as the positive control.

Positive Ki-67 immunostaining appeared as a brown color that was present only within the nucleus. Five high-power fields (HPFs, 400×) were randomly selected from each slide for calculation, and the Ki-67 index is presented as the percentage of mean positive cells. Positive ESM-1 immunostaining appeared as a yellow or brownish yellow color within the cytoplasm. Five HPFs were randomly selected from each slide, and 100 cells were observed in each HPF. The intensity was scored as 0 = negative, 1 = weak, 2 = intermediate, and 3 = strong. The percentage of positive cells was scored as 1, 2, 3, and 4 for ≤25, 26–50%, 52–75%, and ≥ 75%, respectively. The ESM-1 index was calculated as intensity score × percentage of positive cell score, and ESM-1 index scores of 0, 1–2, 3–6, and > 6 indicated negative, weak, intermediate, and strong, respectively. Low and high ESM-1 expression was defined as 0–2 (negative and weak) and ≥ 3 (intermediate and strong), respectively. The high expression rate was calculated using the following formula: number of patients with ESM-1 index of ≥3/total number of patients × 100%.

### Statistical analysis

For categorical variables, comparisons between high and low expression of ESM-1 were performed using the chi-square test. Continuous variables were compared using the independent two-sample *t* test. Descriptive statistics are reported as numbers and percentages for categorical variables or as means with standard deviations (SDs) for continuous variables. All statistical assessments were two sided, and a P of < 0.05 was considered statistically significant. Statistical analyses were performed using SAS (version 9.4;SAS Institute, Inc., Cary, NC, USA).

## Results

The mean age of the patients was 49.0 (SD: 11.45) years, and 54 and 40 patients were women and men, respectively. MRI data were available for all the patients. The average maximum tumor size was 2.8 (SD: 1.0) cm. In addition, tumor invasion was evaluated through MRI examination. Signs of invasion were observed in 46 (47.9%) of the 94 patients (Table 1).

**Table 1:** Patients’ demographics and basic clinicopathological parameters

Characteristics	*N* = 94
Age (years)	49.0 ± 11.45
Gender, n (%)	
Male	40 (42.6%)
Female	54 (57.4%)
Invasion state, n (%)	
Invasive NCA	45 (47.9%)
Non-invasive NCA	49 (52.1%)
Ki-67, n (%)	
< 3%	66 (70.2%)
> =3%	28 (29.8%)
Maximum tumor diameter (cm)	2.8 ± 1.04

*NCA* Null cell adenoma

**Fig. 1:**
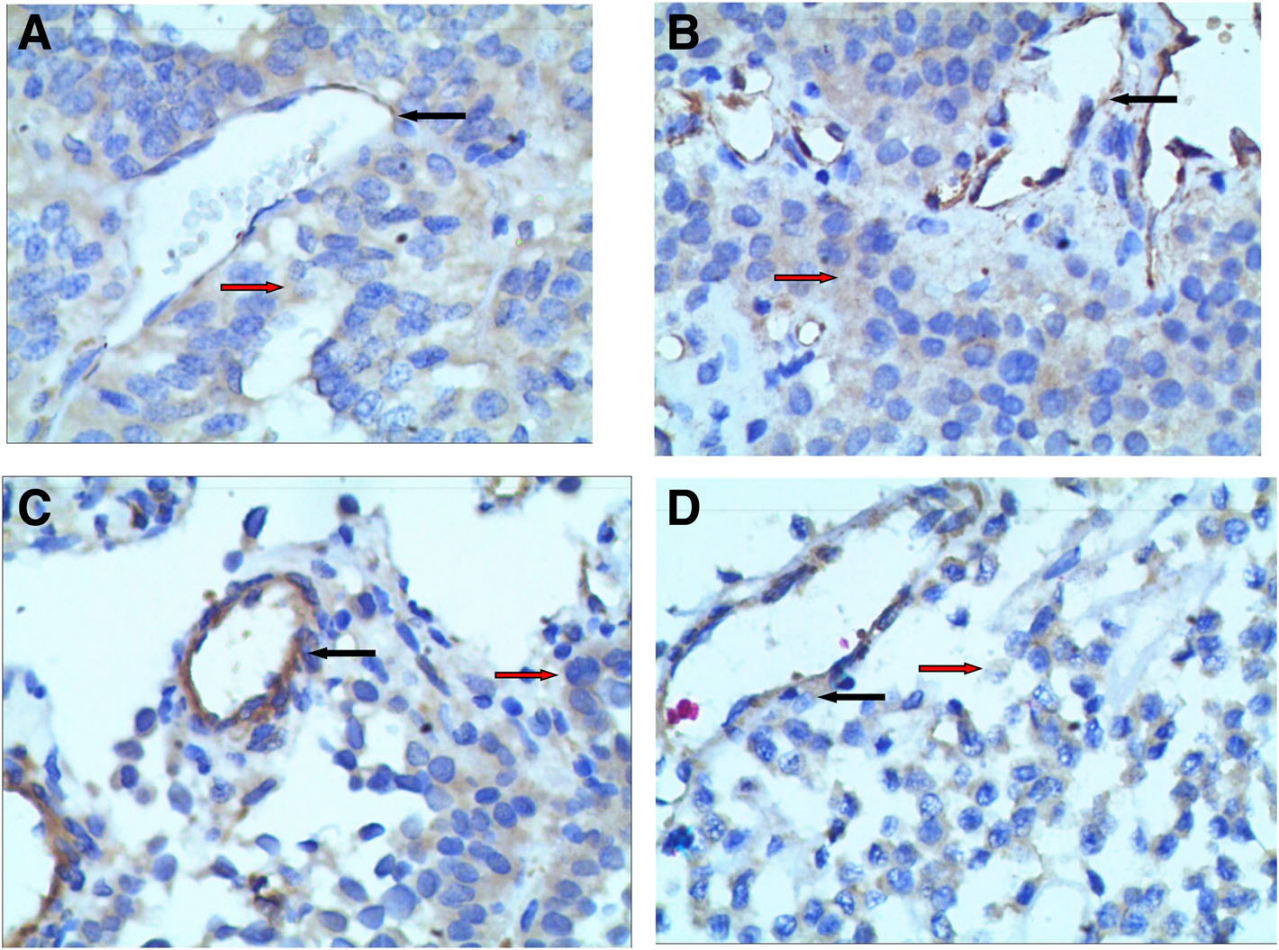
**a** Strong endothelial cell-specific molecule-1 (ESM-1) index was seen in the cytoplasm of vascular endothelial cells, and weak ESM-1 index was seen in the cytoplasm of adenoma cells; **b** intermediate ESM-1 index was seen in the cytoplasm of vascular endothelial cells, and weak ESM-1 index was seen in the cytoplasm of adenoma cells; **c** weak ESM-1 index was seen in the cytoplasm of vascular endothelial cells, and intermediate ESM-1 index was seen in the cytoplasm of adenoma cells; **d** both intermediate ESM-1 index was seen in the cytoplasm of vascular endothelial and adenoma cells. Black arrow: vascular endothelial cells with ESM-1 expression; red arrow: adenoma cells with ESM-1 expression

ESM-1 expression was detected in both vascular endothelial tissues and adenoma tissues. The representative images for ESM-1 expression in vascular endothelial tissues and adenoma tissues are shown in Fig. 1. The high expression rate of ESM-1 in vascular endothelial tissues (63/94, 67%) was significantly higher than that in adenoma tissues (49/94, 52%; *P* = 0.037). Table 2 shows the association between ESM-1 expression in vascular endothelial tissues and clinicopathological parameters. Significant differences were observed in the invasion state and maximum tumor diameter between high and low expression of ESM-1 in vascular endothelial tissues (both *P* < 0.05). Significant positive associations were observed between ESM-1 expression in vascular endothelial tissues and tumor invasion (*P* = 0.002) and tumor size (*P* = 0.020). However, only tumor size was associated with ESM-1 expression in adenoma tissues (*P* = 0.016, Table 3).

**Table 2:** The association between the expression of endothelial cell specific molecule- 1 in vascular endothelial tissue and clinicopathological parameters

Characteristics	Low expression (*n* = 31)	High expression (*n* = 63)	*P*-Value
Age (years)	51.2 ± 11.27	48.4 ± 11.58	0.466
Gender, n (%)			0.932
Male	13 (41.9%)	27 (42.9%)	
Female	18 (58.1%)	36 (57.1%)	
Invasion state, n (%)			0.002*
Non-invasive NCA	22 (71.0%)	23 (36.5%)	
Invasive NCA	9 (29.0%)	40 (63.5%)	
Ki-67, n (%)			0.554
< 3%	23 (74.2%)	43 (68.3%)	
> =3%	8 (25.8%)	20 (31.7%)	
Maximum tumor diameter (cm)	2.5 ± 0.68	3.0 ± 1.16	0.020*

*NCA* Null cell adenoma *: *p* < 0.05

**Table 3:** The association between the expression of endothelial cell specific molecule- 1 in adenoma cells and clinicopathological parameters

Characteristics	Low expression (*n* = 45)	High expression (*n* = 49)	*P*-Value
Age (years)	48.8 ± 11.03	49.1 ± 11.94	0.893
Gender, n (%)			0.370
Male	17 (37.8%)	23 (46.9%)	
Female	28 (62.2%)	26 (53.1%)	
Invasion state, n (%)			0.065
Non-invasive NCA	26 (57.8%)	19 (38.8%)	
Invasive NCA	19 (42.2%)	30 (61.2%)	
Ki-67, n (%)			0.526
< 3%	33 (73.3%)	33 (67.3%)	
> =3%	12 (26.7%)	36 (32.7%)	
Maximum tumor diameter (cm)	2.5 ± 0.87	3.1 ± 1.13	0.016*

*NCA* Null cell adenoma *: *p* < 0.05

## Discussion

The results of the present study indicated that in pituitary NCA, even ESM-1 expression was observed in both vascular endothelial and adenoma tissues, a significant positive association between tumor invasion and ESM-1 expression was observed only in vascular endothelial tissues, suggesting that the progression of tumor occurs mainly through the vascular structure via ESM-1-mediated mechanism is involved.

Pituitary adenoma is a common pathological change accounting for approximately 10% of intracranial tumors and is frequently asymptomatic and benign. Clinically pituitary adenoma are classified as functional and nonfunctional, and nonfunctional pituitary adenomaaccount for 30% of all pituitary adenoma [23, 24]. Heterogeneity is a unique feature of nonfunctional pituitary adenoma [24]. The association between the tumor invasion of pituitary adenomas and the expression of ESM-1 has been confirmed in several studies [14, 18, 24]; however, the source of functional ESM-1 production and secretion for tumor invasion remains unknown. Matano et al. found that ESM-1 was expressed in more than 90% of CD34^+^ vascular endothelial cells [19]; however, Maio et al. reported that ESM-1 expressed by adenoma cells can reflect invasion or progression more accurately, meanwhile ESM-1 expression by vascular endothelial cells cannot [14]. Both Matano et al. and Miao et al. have used the Knosp grade [21] to estimate tumor invasion in specimens obtained from patients with pituitary adenoma who underwent transsphenoidal surgeries. These inconsistent results might be attributable to the highly heterogeneous characteristics of pituitary adenoma. Several classifications exist within nonfunctional pituitary adenomas [20], and if functional pituitary adenoma are additionally included, the composition of investigated specimens may become too complicated and heterogenous to make a clear conclusion. Thus, we selected only NCA in this study because we believe that analyzing different types of pituitary adenoma separately can solve this problem. A better understanding can be obtained and a consensus can be reached for pituitary adenoma when results from other cell types would be available. However, we have to mentioned one important limitation that since the date of sample collection was before 2017, the release date of the 4th edition of the WHO classification of endocrine tumors [25], the classification of NCA is base on the 2004 edition. Using the 4th edition of the WHO classification for NCA, immunonegativity for pituitary transcription factors and adenohypophyseal hormones are necessary, however we did not have complete results of these assays, neither the enough amounts of specimens from all the included patient to do these assays; so we still follow the 2004 edition [20].

The relationship among angiogenesis and ESM-1 expression in pituitary adenoma is still unclear. The expression of VEGF, the key regulator of angiogenesis was not evaluated in our study due to limited available data and specimens. The expression of other important angiogenetic molecules, including FGF, stromal cell-derived factor, and the MVD was not detected. The possibility of ESM-1 doesn’t not involve in angiogenesis cannot be ruled out by our results. However, the invasiveness of tumor is certainly associated with the expression of ESM-1 via vascular structure.

Although pituitary adenoma are mostly benign, their invasiveness remains a concern because 35% of them become invasive and can invade adjacent structures, including cavernous and sphenoid sinuses. Because nonfunctional pituitary adenoma do not cause hormone hypersecretion, they are difficult to diagnose because they are asymptomatic in the early stage; the diagnosis is often established when nonfunctional pituitary adenoma increase in size and become invasive. Invasiveness is a prominent factor for surgical curability, since surgical approach is the only available treatment of nonfunctional pituitary adenoma now, invasiveness relates to patients’ outcome, too [26, 27]. We used multiple indexes, namely the Knosp grade and Wilson–Hardy classification with or without extrasellar extension, to define invasion; because these methods still require a degree of subjective judgments, we combined these methods to minimize the effects. Our finding of a significant association of vascular ESM-1 expression with invasion might be used as a more objective marker to evaluate the invasiveness of pituitary adenomas; this method would require less resources and instruments and can be widely used in many institutes. In addition, this finding can be a useful predictor of recurrence. We used immunohistochemistry rather than RT-PCR to detect ESM-1 expression. In the first study of ESM-1 expression in pituitary adenoma conducted by Cornelius et al., immunohistochemical staining was used to identify specific cell types or location of ESM-1 expression [18].

The maximum diameter of a pituitary tumor before surgery is a predictive factor of recurrence because a larger tumor may be less accessible during surgery [28]. ESM-1 expression was reported to be associated with a larger preoperative tumor size [18]. This finding is in agreement with our result that ESM-1 expression in NCA was significantly associated with tumor size both in vascular endothelial and adenoma tissues. However, we found that ESM-1 expression in only vascular endothelial tissues was associated with tumor invasion, suggesting that angiogenesis may be one of the mechanism underlying invasion in NCA, similar to other types of tumors [3–6]. Because a study reported that CD34/CD105 MVD was not correlated to invasion represented by Knosp grades and that angiogenesis may not be the primary driver of ESM-1-mediated pituitary tumor invasion [14], the real association and mechanism underlying ESM-1-mediated NCA invasion could be analyzed by detecting several crucial factors of angiogenesis. Heterogeneity should be considered in future research. In different types, the mechanism and key molecules of angiogenesis might be completely different, and the effect of angiogenesis on invasion might vary.

Both preoperative and postoperative tumor size are also related to the outcome of patients with nonfunctional pituitary adenoma who underwent transsphenoidal surgery. Transsphenoidal surgery is still the first-line treatment of nonfunctional pituitary. Assessment of the change of tumor remnats is important for predicting patients’ outcome. Spontaneous shrinkage of the tumor remnants after transsphenoidal surgery for 3 to 6 months was occured in 40 to 50% of patients in previous studies [29, 30]. Spontaneous decreases in the volume of nonfunctional pituitary adenoma without intervention has also been observed in the studies of the natural course of incidentalomas [31]. Small preoperative tumor volume, small craniocaudal tumor remnants are positively associated with spontaneous shrinkage of the tumor remnants [29]. We found that significant positive associations were noted between tumor size and both ESM-1 expression in vascular endothelial tissues and in adenoma tissues. ESM-1 expressed in the two types of tissue may both related to the change of tumor size. Furthermore, dynamic MRI evaluation indicated that the blood supply pattern, the ascending pattern is another positive predictor of sponteouous shrinkage of tumor remnants. Ascending pattern means that the arterial blood is supplied in the caudal to rostral direction and no regression was found in the tumor remnants with rich blood supplies [30]. The reverse direction pattern, the descending pattern is found in the normal pituitary gland. The mechanisms of the changes of the direction and the amount blood supplies of pituitary adenoma remains unknown, we believe the changes of microcirculation of the tumor may play a role, ESM-1 may involve possiblily via structural regulation ie angiogenesis, or functional regulation, for example the vasodilation controlled by biochemicals. Our study of ESM-1 is preliminary and more studies are required to clarify the changes of tumor circulation environment in the future.

Similar to the finding for Ki-67, reported by Cornelius et al., we could not find a significant association between ESM-1 and Ki-67, although a Ki-67 index of > 3% is considered a prognostic factor in pituitary tumors [18, 32]. Ki-67 was not associated with VEGF and MVD [12] and may not be associated with angiogenesis in pituitary adenoma. The possibility of using ESM-1 as a recurrent predictor should be examined in future studies.

This study has some limitations. The sample size of our study is still relatively small; the recurrence of these patients should be followed up to investigate the relationship between ESM-1 expression and patients’ outcomes. The mechanism underlying ESM-1-mediated invasion in NCA should be investigated in the future.

## Conclusions

In this preliminary study, we found a significant positive association between tumor invasion and ESM-1 expression was observed only in vascular endothelial tissues, suggesting that tumor progression occurs mainly through ESM-1-associated mechanism in NCA. The precise role of ESM-1 in the regulation of tumor invasion should be investigated to clarify whether the structural or the functional regulation of tumor circulation environment involves, in addition, the actual stage of NCA in which ESM-1 affect also has to be evaluated in the future.

## Data Availability

The datasets used and/or analysed during the current study are available from the corresponding author on reasonable request.

## Abbreviations

ESM-1: Endothelial cell-specific molecule-1
FGF: Fibroblast growth factor
HRP: Horseradish peroxidase
MRI: Magnetic resonance imaging
MVD: Microvessel density
NCA: Null cell adenoma
VEGF: Vascular endothelial growth factor
